# Spatiotemporal distribution of green-certified buildings and the influencing factors: A study of U.S.

**DOI:** 10.1016/j.heliyon.2023.e21868

**Published:** 2023-10-31

**Authors:** Siwei Chen, Zhonghua Gou

**Affiliations:** School of Urban Design, Wuhan University, Wuhan, China

**Keywords:** Green building, Spatiotemporal distribution, Energy policy, Geographical and temporal weighted regression, Demographic and socioeconomic factors

## Abstract

Green building development is a global strategic plan aimed at addressing environmental burdens and reducing energy consumption in the building sector. Currently, research does not adequately reveal the spatiotemporal patterns of green-certified building development and the factors that influence it. To address this gap, this study investigates the dynamic distribution of Leadership in Energy and Environmental Design (LEED) certified projects in the U.S. by incorporating time effects into spatial regression models. The results reveal that (1) significant regional variations in the spatiotemporal distribution of green-certified buildings (global Moran's index for 2017, 2019 and 2021 are 0.0172, 0.0327 and 0.0622 respectively). (2) Demographic, socioeconomic, environmental, and policymaking factors explain the observed patterns (the mean values of the coefficients of population size, the Caucasian demographic proportion to the total population, income inequality, regional price parity, and average annual temperature were 8236.1383, −18.9113, −533.1024, 365.1813 and 227.1735 respectively). (3) Expedited permitting, reduced fees, and property tax credit or exemption (p-values less than 0.01) are significant policy instruments that promote the implementation of LEED certified projects. The findings offer pivotal insights that enable targeted interventions, informed decisions, and effective resource allocation. Furthermore, it furnishes a reference for strategically siting green building initiatives in the next phase, encompassing zero-energy buildings, green technologies, and low-carbon solutions. Enhancing understanding of complexities in U.S. green-certified building practices, this research acts as an evidence-based cornerstone across sectors. Urban planners can leverage these insights to allocate resources efficiently and steer green-certified projects, for impactful environmental sustainability and community progress. Policymakers can customize incentives based on drivers of adoption, promoting equitable distribution. Meanwhile, construction stakeholders can optimize strategies through decoding temporal and spatial adoption patterns, leading to prudent resource use and project success.

## Highlights

• This study thoroughly investigates the spatiotemporal dynamics of green building adoption.
• It illuminates temporal and spatial variations of green buildings across different building types and certification levels.
• The geographically and temporally weighted regression model elucidates the spatiotemporal heterogeneity.
• Expedited permitting, reduced fees, and property tax credit or exemption are important.
• Policy recommendations based on the spatiotemporal heterogeneity and influencing factors.

## Graphical abstract

**Figure:**
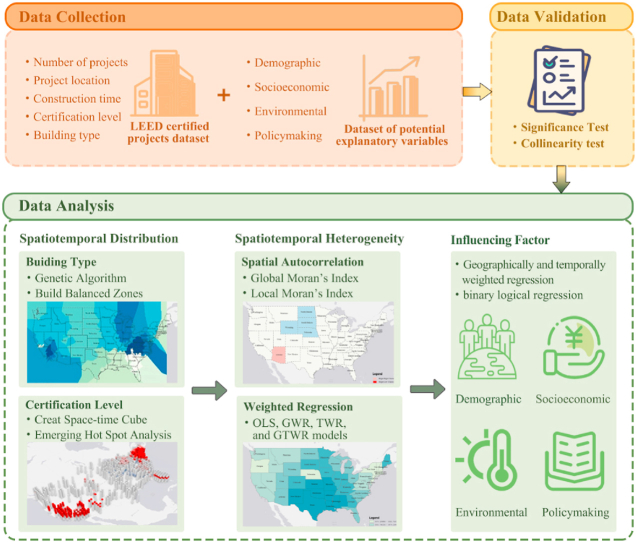

## 1 Introduction

The construction industry is a significant contributor to energy consumption and greenhouse gas emissions, responsible for the greatest proportion of environmental load [1]. In particular, energy consumption in buildings accounts for 40 % of global energy consumption and 30 % of global greenhouse gas emissions [2]. With both soft and hard incentives such as subsidies and regulations, transitioning and upgrading to green buildings has become one of the dominant modes of development for the building sector [3,4]. Green building development has become a strategic requirement for most countries worldwide [5]. Large-scale green building development has the potential to minimize the negative impacts of residential and commercial buildings on the environment, the ecology, and their occupants while achieving carbon neutrality goals meanwhile reducing the overall life cycle costs of the project. The financial significance of green building development, valued at trillions of dollars, is driving interest in green building applications worldwide [6]. As a result, there is a growing demand for green building applications in buildings worldwide, and green buildings are gaining in popularity [7]. To address this trend, green building rating systems have emerged, aiming to provide standardized assessment and certification for various types of constructions. Globally, the landscape of green building rating systems has expanded significantly to encompass over 600 distinct frameworks [8]. While BREAM (Building Research Establishment Environmental Assessment Method, United Kingdom) and LEED (Leadership in Energy and Environmental Design, United States) maintain prominent positions as widely recognized certification schemes, other systems including DGNB (Deutsche Gesellschaft für Nachhaltiges Bauen, Germany), CASBEE (Comprehensive Assessment System for Built Environment Efficiency, Japan), and ASGB (Assessment Standard of Green Buildings, China) have gained substantial adoption across various regions [9]. They encompass a spectrum of criteria extending beyond energy efficiency to address indoor air quality, water management, and social well-being [10]. Regular updates to these systems reflect a commitment to align with evolving knowledge and best practices in the field. As cities and societies continue to emphasize sustainability and responsible development, these rating systems offer objective tools for guiding architectural and construction decisions.

In recent years, with the continuous expansion of the green building market, a large number of studies have been devoted to exploring the geographic distribution of green buildings in an effort to identify the spatial characteristics and distribution patterns of green buildings across countries, regions as well as on the scale of cities. For example [11], used spatial autocorrelation analysis to find that there is an obvious aggregation of county-level green buildings in the United States, showing a pattern of regional imbalance. This distribution pattern is also present in China [12]. There are also some studies that explore the types of regions where green buildings are more prevalent to be implemented. For example, while factors like population, regional activities, and professional organizations contribute, cities implementing LEED-based policies have driven greater green-certified building growth [13]. [14] found that LEED-certified projects are implemented more frequently in communities with lower densities of vulnerable populations in the cities of Austin and Chicago in the United States [15]. found that China's green buildings are significantly more prevalent in the cities with more developed economies than in neighboring cities [16]. focused on LEED-certified multifamily buildings, and the findings suggest that this type of green building was more favorably distributed in areas with higher income levels [17]. highlighted the considerable influence of evolving urban planning guidelines on the selection of sustainable sites. This contributes to obtaining third-party certifications, particularly LEED certification, thus exerting a substantial influence on the developmental trajectory of two generations of new cities in Egypt. Additionally, in studies by Refs. [18,19], it was demonstrated that regional cooperation and pilot and demonstration projects accelerated the distribution of green buildings in cities. This is because regional cooperation significantly enhances the efficiency of green building promotion, especially in developed areas, due to the spatial correlation observed in green building construction. Pilot and demonstration projects expedite the early dissemination of new technologies, refining green building standards. These projects lower future adopters' costs, thereby accelerating green building adoption. In general, the distribution of green-certified buildings exhibits a clear clustering tendency, yet variations exist within specific countries, regions, and cities. Political and economic factors are regarded as common influencing factors contributing to these distribution disparities.

Many drivers and government initiatives have been proved to have an impact on the distribution of green buildings, and understanding these factors and initiatives is particularly essential to promote the implementation of green buildings in cities. Therefore, several studies have explored the significance and interactions of the influencing factors from different perspectives. For example [20], argued that regional economy is the most critical and fundamental factor leading to the spatial distribution of green buildings, and explored the influence of economic factors on the spatial distribution of green buildings in Guangdong Province, China, using a gray correlation model. While [21] used a fuzzy synthetic evaluation method and found that it is the environmental impact that is the most critical factor influencing the distribution of green buildings in Vietnam. In Li et al. (2018) study, the anchoring effect of behavioral economics was introduced to provide a new perspective focusing more on demographic factors, and it was found that less educated populations are more likely to be affected by price anchoring and more dependent on external information, which in turn affects the implementation of green buildings. Some studies have also shown that government policy and regulatory instruments have a significant positive impact on green building adoption, such as subsidy, expedited permitting [22,23].

Given the large-scale implementation of green buildings in many countries, the research findings on the spatiotemporal distribution of green buildings are limited. Few pieces of literature have included temporal factors in their studies, and in-depth analyses using geographic information technology and spatiotemporal statistical methods are still lacking [18,24]. Therefore, to fill the research gap, this study investigates the distribution of green-certified buildings from a spatiotemporal perspective by considering the level of development (demographic, socioeconomic, and environmental) in different regions and exploring its key influencing factors. It also discusses the impact of implementing various policy instruments. The United States, with extensive expertise in green-certified building development [25], predominantly adopts the Leadership in Energy and Environmental Design (LEED) certification system by the United States Green Building Council (USGBC). This system is the preferred choice for green building certification in the United States and enjoys significant international adoption [26,27]. As of the conclusion of 2022, a total of 116,999 LEED projects were registered, with 70,706 of these achieving LEED certifications in the United States. Notably, this study employs the terms “green-certified building” and “LEED project” interchangeably, acknowledging the prominence of LEED projects within the realm of green-certified buildings in the United States. Therefore, this study focuses on LEED certified projects in the United States to investigate their spatiotemporal distribution and the factors influencing these distributions.

Novelties and contributions of this study are as follows: (1) Incorporating temporal effects into spatial regression models. Based on three specific dimensions: demographic, socioeconomic, and environmental, the study explores the factors influencing the spatiotemporal distribution of LEED projects in the United States and explains the trends of each variable based on the geographically and temporally weighted regression (GTWR) model in both temporal and spatial dimensions. (2) Adopting a statistical approach to explore the influence of policymaking factors on the implementation of LEED projects in the United States. It identifies which policy instruments are more efficient to use in different regions, given varying levels of social development. Not only will the results of this study help policymakers gain a better understanding of green-certified building practices in different regions, but also assist them in developing locally appropriate policies to encourage the development of more green-certified buildings. It will also provide more efficacious guidance for the next stage of green buildings, such as ultra-low energy buildings, near-zero energy buildings, and zero energy buildings. And contributes to optimizing the siting of green and low carbon technologies such as green roofs, green walls, photovoltaic panels, and heat pumps and promotes their more equitable and extensive distribution in cities.

## 2 Methodology

### 2.1 Data collection

This study takes the 50 states of the United States as the study area. It uses a state-level panel dataset from 2000 to 2021 to explore the spatiotemporal distribution and influencing factors of LEED certified projects. Data were collected from various sources from November to December 2022. There are two parts to the dataset. The first part concerns the location, number, certification year, certification level, and building type of LEED projects in the United States. Detailed information on the number of LEED projects is provided in Table S1 in the Supplementary Material. Figs. S1 and S2 in the Supplementary Materials illustrate further the differences in the proportion of LEED projects in terms of building types and certification levels between different states. The second part is the data used to explain the heterogeneity in LEED projects. These data are divided into four subcategories: Demographic data (including population size, population density, percentage of the total population by ethnicity, percentage of population ages 65 and above, and international migration rate); Socioeconomic data (including educational attainment, life expectancy, infant mortality, real gross domestic product (GDP) per capita, median household income, income inequality index, regional price parity, unemployment rate, and human development index); Environmental data (including annual precipitation, average annual temperature, cooling degree days, heating degree days, mean elevation, and distance to the nearest coastline); and Policymaking data (including the content of policies, operative provision, and policy instruments).

In the spatiotemporal heterogeneity analysis, the dependent variable in the weighted regression model is the number of LEED certified projects by states in 2017, 2019, and 2021 (including the four ratings of Platinum, Gold, Silver, and Certified) because LEED certified projects in the United States began to show heterogeneity after 2017. The independent variables come from the demographic, socioeconomic, and environmental variables collected in the second part of the dataset, which is regarded as potential explanatory variables. The descriptions, sources, and references of the potential explanatory variables are provided in Supplementary Material Table S2. In exploring the impact of policymaking factors on LEED certified projects, the independent variable for the binary logistic regression was whether or not the following eight policy instruments were adopted. Descriptions and examples of the policy instruments are presented in Table 1. The dependent variable is the number of LEED certified projects in each state. Using the natural breakpoint method, the number of LEED certified projects was divided into three categories: high, medium, and low.

**Table 1:** Description of policy instruments and example policies.

Policy Instrument	Description	Example Policy	
		**Policy Title**	**Operative Provision**
Requirement	Require certain buildings to earn LEED certification.	Green Buildings Action Plan, Executive Order B-18-12	All new and majorly renovated state buildings larger than 10,000 square feet are required to earn LEED silver certification.
Enabling or Encouraging Legislation	Encourage buildings to earn LEED certification or follow LEED standards	Resolution 3136–06	Encourage all commercial and institutional projects to follow a LEED checklist and to seek LEED certification.
Expedited Permitting	LEED certified buildings may qualify for expedited building plan review	Seattle Priority Green Expedited Permitting Program	Provide priority scheduling and expedited initial review and permitting to commercial and residential projects that earn LEED gold or platinum certifications.
Fee Reduction	Generally including building permit fee, certification fee, land development application fee and urban sales tax	Public Act 13–308	Development fee reductions for projects that achieve LEED gold on brownfield sites.
Density or Height Bonus	LEED certified buildings are eligible for additional density or height	Ordinance 9–6.407	Private developments that earn LEED gold or platinum certifications are eligible for residential density, floor area ratio, and height bonuses.
Property Tax Credit or Exemption	LEED certified buildings are eligible for property tax credit or exemption	New York Chapter 188, SB 1462	Ten-year property tax exemption for green buildings and construction improvements that meet LEED certification standards.
Marketing or Technical Assistance	LEED certified buildings are eligible for marketing benefits or technical guidance	HB 255	Provide technical assistance to local governments, area development districts, housing authorities and school districts to maximize energy savings and efficiency.
Financing	LEED certified buildings are eligible for funding	New York Green Residential Building Program, SB 8134-B	Create a green residential building grant program to encourage compliance with green building standards and criteria based on LEED for Homes.

### 2.2 Data validation

A Pearson's correlation coefficient test is necessary to validate the quality of the demographic, socioeconomic, and environmental data in the second part of the dataset and ensure non-linear relationships between these explanatory variables. Table S3 in the Supplementary Material shows five variables that passed significance and collinearity tests: population size, Caucasian demographic proportion, income inequality, regional price parities, and average annual temperature. Table S3 also provides descriptive statistical data for these five explanatory variables to characterize the statistics and verify the veracity of the dataset. Fig. S3 in the Supplementary Materials illustrates the Pearson correlation coefficient matrix with p-values below 0.05 and correlation coefficients below 0.7 for 2017, 2019, and 2021, as shown in the Supplementary Material. This study, conducted in 2022, utilizes data collected up to the previous full calendar year (2021). Acknowledging the heterogeneity observed in U.S. LEED certified projects since 2017, this research adopts a biennial framework, focusing on the years 2017, 2019, and 2021 as the temporal scope of analysis. The correlation coefficients range from [−0.56,0.62], and the variance inflation factor (VIF) values of each variable are less than 3, indicating that these explanatory variables are not highly correlated in the original dataset and there is no multicollinearity. Therefore, they can be used for spatiotemporal heterogeneity analysis.

### 2.3 Data analysis

#### 2.3.1 Several analytical methods used in this study are described below

Genetic algorithm (GA) has been widely used for optimization problems in spatial planning due to its feature of searching for optimal global solutions [28]. More details and pseudo-codes of GA can be found in previous studies [29,30]. In this study, GA is applied to the problem of site allocation for LEED certified projects with different building types. Using the Build Balanced Zones tool in ArcGIS Pro to create spatially contiguous zones as compact as possible based on GA, where each zone consisted of approximately the same number of elements.

Using Space-time cube analysis, cells were created within the study area [31]. Cells were separated by a distance interval of 100 km × 100 km, with a time step interval of 1 year. Each cell contains panel data for LEED projects with different certification levels between 2000 and 2021. The emerging hot spot analysis tool is then used to identify the cold and hot spots of LEED certified projects at different spatiotemporal locations by Mann-Kendall statistics and analyze the characteristics of cold and hot spots over time [32]. The 3D visualization space-time cube tool (one of the effective techniques to detect spatial and temporal trends and patterns) was then used to explore the spatiotemporal trends of cold spots and hot spots with statistical significance [33].

From a nationwide perspective, global spatial autocorrelation is applied to assess the overall patterns and trends in the spatial distribution of LEED certified projects in the study area. Moran's Index is a crucial indicator of global spatial autocorrelation, which measures the degree of aggregation of LEED certified projects in the study area and assesses whether the overall pattern is clustered, dispersed, or random. Given that a certain degree of spatial dependence or variation between states is common [34], this study also used local spatial autocorrelation to detect statistically significant hot spots, cold spots, and spatial outliers.

Ordinary least squares regression (OLS) model is an inferential technique used to regress the dependent variable on other explanatory variables. It is a technique that assumes the existence of spatially stationary and constant relationships. Specifically, the weighting calculations of OLS models ignore spatial location information, and thus information about spatial heterogeneity may be lost [35].

Geographically weighted regression (GWR) models, compared to OLS models, are linear regressions used to simulate local spatially varying relationships. Furthermore, it follows entirely the first law of geography, which enhances the science and validity of the model, particularly in the fields of geography and meteorology [36]. Temporally weighted regression (TWR) models are another technique for modeling varying temporal relationships compared to OLS models [37]. Because GWR models cannot handle temporal varying of parameters, Geographically and temporally weighted regression (GTWR) models capture the spatial and temporal heterogeneity on the weighting matrix of spatial and temporal dimensions when dealing with parameters. Therefore, the GTWR model regression results are more accurate and reliable [38].

This study also used binary logical regression to explore the relationship between LEED project implementation and the eight policy instruments mentioned above in the LEED-related policies. The response scale was simplified to “yes = 1″ and “no = 0″ before applying the regression analysis [39]. Preliminary experiments were conducted to select relevant policymaking variables and to exclude other parameters from the regression. All data are presented with 95 % confidence intervals and a significance level set at 0.05.

Overall, this study encompassed several steps. Firstly, two databases were established—one for LEED-certified projects and another containing potential influencing variables. Afterward, the data underwent significance and collinearity tests. Subsequently, balanced zones were created using a genetic algorithm to explore the spatial distribution of different types of LEED projects. Space-time cubes were formed, and an emerging hot spot analysis tool investigated spatiotemporal patterns of various LEED certification levels. The Global and Local Moran's Index examined project heterogeneity, followed by a comparison of model performance between OLS, GWR, TWR, and GTWR models. The GTWR model was then employed to analyze the influence of demographic, socioeconomic, and environmental factors on the distribution of LEED projects. Additionally, a binary linear regression model examined the significant impact of different policy instruments on the implementation of LEED projects. Ultimately, the study summarized the spatiotemporal distribution patterns of LEED projects, elucidated the mechanisms behind influencing factors, and proposed policy recommendations.

**Fig. 1:**
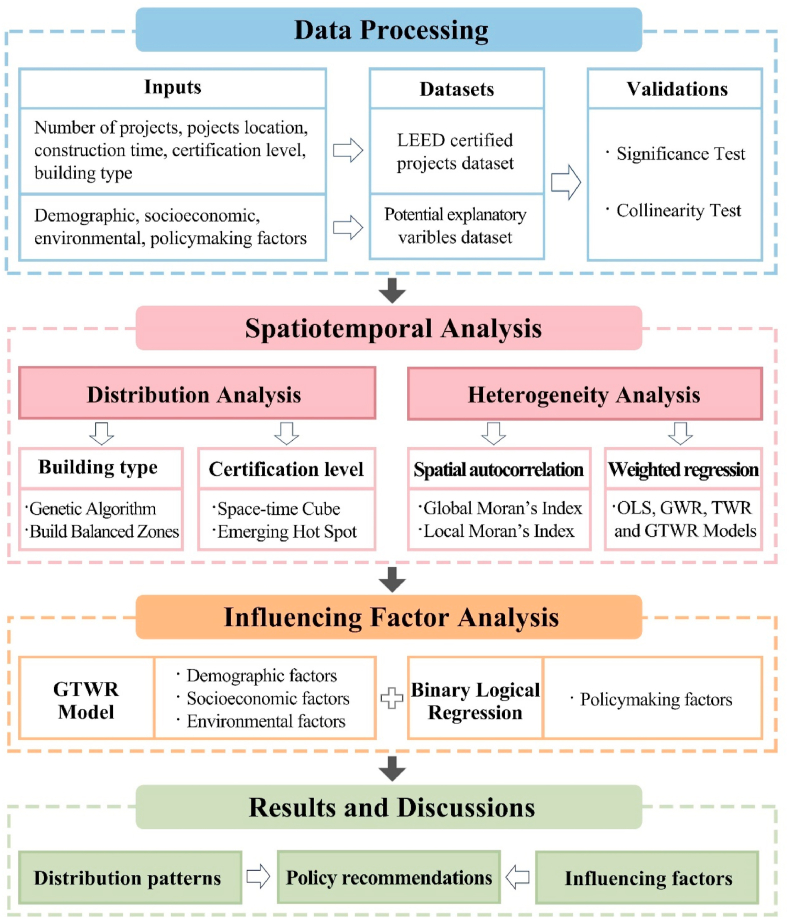
The research framework of this study.

Further details and formulas for each method can be found in the Supplementary Materials. A specific flowchart based on these methods is shown in the Graphical Abstract and Fig. 1.

## 3 Results

### 3.1 Results of spatiotemporal distribution analysis

#### 3.1.1 LEED project distribution by building type

**Fig. 2:**
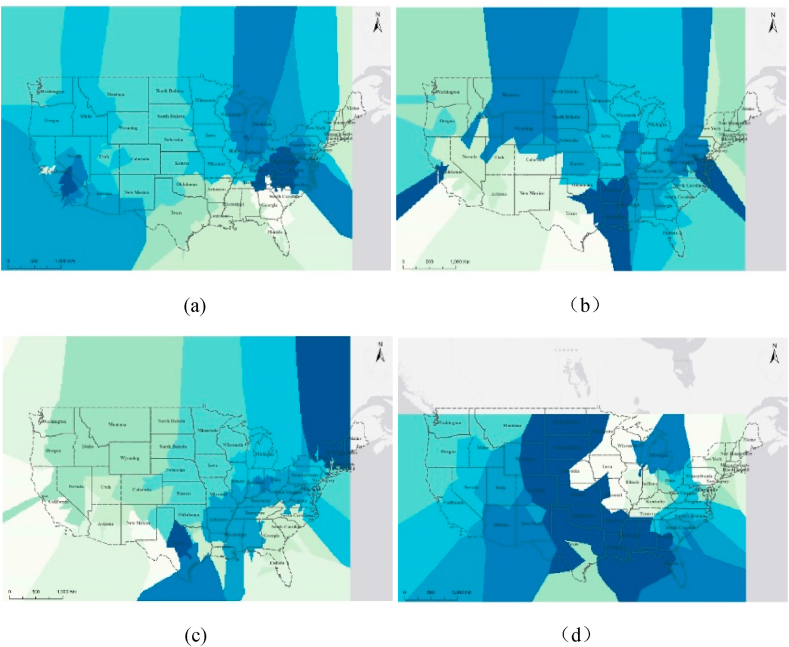
Spatial distribution of LEED projects of different building types (the color scheme employed in these figures solely represents different zones without any substantive meaning). (a)commercial building; (b)office building; (c)residential building; (d)education building.

In this study, LEED certified projects are divided into four main categories by building type: residential buildings, commercial buildings, office buildings, and educational buildings. Fig. S1 in the Supplementary Materials reveals that the vast majority of LEED-certified projects in the states favor residential buildings, with Texas in particular having a large number (n = 6408) and 92.51 % of residential LEED projects. Meanwhile, LEED-certified projects also have a higher distribution in commercial buildings (20.27 %). The spatial distribution characteristics of LEED certified projects across these four building types are revealed in Fig. 2. A different color distinguishes each zone, and the number of LEED certified projects within each zone is the same. The larger the zone, the smaller the number of LEED certified projects contained within the zone, and the lower density of LEED certified projects. The results show that most commercial buildings are located in California (20.66 %) and along the eastern coast, with significant aggregations near the coastline in Illinois and Indiana. Inland regions such as North Dakota (0.10 %), South Dakota (0.14 %), and Nebraska (0.27 %) have a lower distribution density. Office buildings are more densely distributed along the coastline than in inland areas. Residential buildings are particularly abundant in Texas (25.11 %), while very few are in North Dakota (0.02 %), South Dakota (0.01 %), Nebraska (0.01 %) and Montana (0.04 %). Educational buildings are heavily distributed in Ohio (22.21 %), Illinois (7.89 %), and Maryland (7.89 %), with an overall spatial pattern of greater density in the east than in the west.

#### 3.1.2 LEED project distribution by certification level

LEED projects are rated into four levels, low to high, LEED-certified, silver-certified, gold-certified, and platinum-certified. Fig. S2 in the Supplemental Materials visualizes the differences between certification levels in different states. For example, Texas has an exceptionally large number of LEED-certified green buildings (78.06 %), whereas the percentage of platinum-certified green buildings is very low (1.29 %). On the other hand, in Idaho, the highest percentage of green buildings is platinum-rated (34.82 %), while the lowest percentage is those just certified (10.12 %).

**Fig. 3:**
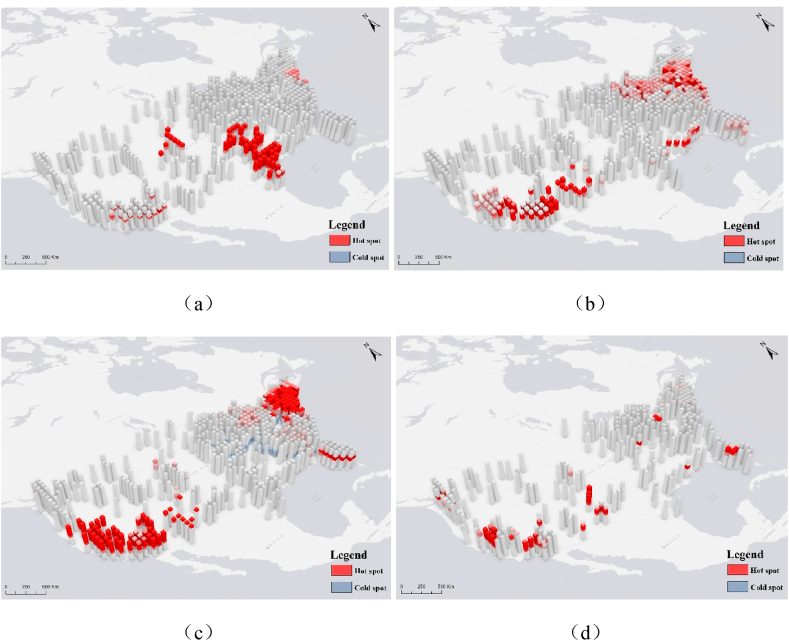
Spatiotemporal distribution of LEED projects with different ratings. (a) LEED-certified; (b) silver-certified; (c) gold-certified; (d) platinum-certified.

Given the enormous number of LEED certified projects (more than sixty thousand projects have been certified as of 2021), it is necessary to analyze their spatiotemporal distribution characteristics so that the distribution trends of LEED projects with different ratings can be explored. As shown in Fig. 3, each cube from bottom to top represents the temporal distribution of LEED projects from 2000 to 2021. Cooler colors represent cold spots, indicating a decreasing trend in the number of distributions; warmer colors represent hot spots, indicating an increasing trend. According to the trend analysis results of the space-time cube, the hot spots of LEED-certified projects have been mainly found in Texas and Colorado in recent years. The spatiotemporal hot spots for silver-certified and gold-certified LEED projects show roughly the same pattern. Emerging hot spots are located near the eastern and western coastlines, represented by California, New York, New Jersey, and Pennsylvania. However, gold-certified projects emerged as cold spots in the early period of green-certified building construction. They are distributed in the western region of the United States, such as Illinois, Indiana, North Carolina, South Carolina, Virginia, and Georgia. Fewer platinum-certified projects are distributed, and emerging hot spots exhibit a patchy distribution.

### 3.2 Results of spatiotemporal heterogeneity analysis

This study conducted a spatiotemporal heterogeneity analysis to investigate the factors influencing the production of such a distribution pattern of LEED certified projects in the United States.

#### 3.2.1 Spatial autocorrelation analysis

This study used global spatial autocorrelation and local spatial autocorrelation for spatial correlation analysis of LEED certified projects in the United States. The Global Moran's Index assesses the overall patterns and trends in the distribution of LEED certified projects. At the same time, the Local Moran's Index mainly reflects local similarities and differences in the distribution of LEED certified projects between neighboring states.

Table S4 in the Supplementary Material presents the results of the Global Moran's Index for 2017, 2019, and 2021. The p-values for all three years are less than 0.05 and pass the significance test. This implies that the distribution of LEED certified projects in the United States after 2017 has a global spatial correlation, showing a pattern of spatially clustered distribution.

To further explore the characteristics of the clustering effect, the Local Moran's Index was used to assess the local spatial correlation. The clustering and outlier results are shown in Supplementary Material, Figure S4. In 2017, Nevada showed an outlier that presented a low-high cluster. This indicated that California (14.01 %), Arizona (2.17 %), and Oregon (2.20 %) had a higher distribution of LEED certified projects, while Nevada (0.81 %) had a lower distribution of LEED certified projects. Low-low clusters appeared in South Dakota (0.16 %) and Kansas (0.38 %), showing a lower distribution of LEED certified projects in those two states. In 2019, the low-low cluster increased, meaning that North Dakota (0.10 %), South Dakota (0.15 %), and Nebraska (0.15 %) still had a lower distribution of LEED certified projects. By 2021, the low-low cluster range was moving westward, and there were still relatively few LEED certified projects in those states. However, a high-high cluster emerged in Arizona, demonstrating that Arizona (3.87 %), California (13.26 %), New Mexico (3.15 %), and Colorado (3.55 %) distributed more LEED certified projects than neighboring states.

Therefore, according to the results of the Global Moran's Index and Local Moran's Index, there is significant spatiotemporal heterogeneity in the distribution of LEED certified projects in the United States from 2017 to 2021.

#### 3.2.2 Weighted regression analysis

Because LEED certified projects in the United States are spatially and temporally heterogeneous, the geographically and temporally weighted regression (GTWR) model is appropriate for analyzing the spatiotemporal evolution patterns and mechanisms of LEED certified projects. To validate the applicability and accuracy of the GTWR model, ordinary least squares regression (OLS), geographically weighted regression (GWR), and temporally weighted regression (TWR) models were constructed simultaneously to compare and analyze the test results and find the best explanatory model for the spatiotemporal evolution mechanism of LEED certified projects.

The regression results of the four models are shown in Table 2. R^2^ and Adjusted R^2^ reflect the degree of model fit, the residual sum of squares (RSS) indicates the accuracy of the model, and the Akaike information criterion (AICc) information can be used as another important criterion to evaluate the goodness of fit of the models. If the difference in AICc values between different models exceeds 3, it indicates a significant difference between the models. As can be seen from Table 2, compared to the R^2^ of the OLS model (0.7643), the R^2^ of the GWR and TWR models improved to 0.7891 and 0.8427, and the GTWR model produced a higher R^2^ (0.8742), demonstrating that the GTWR model had the highest explanatory power. From the residual sum of squares (RSS), it can be seen that compared to OLS, GWR takes into account the spatial attributes of the LEED certified project distribution, and the RSS is reduced by 25,525,059. The RSS of the GTWR model is significantly lower than that of the GWR and TWR models, proving that the GTWR model has a minor error. The AICc values of the GTWR model are lower than those of the OLS, the GWR, and the TWR models. All of the decreasing values are far above the critical value of 3, which means that the improvement of the models is significant, and the GTWR model is the most optimal one to implement.

**Table 2:** Comparison of goodness of fit for OLS, GWR, TWR and MGWR models.

Model	R^2^	Adjusted R^2^	RRS	AICc
OLS	0.7643	0.7560	91413859.5638	2435.7224
GWR	0.7891	0.7817	65888800.9705	2376.6351
TWR	0.8427	0.8373	60984265.7280	2365.03226
GTWR	0.8742	0.8698	34850387.8198	2281.0995

In summary, the GTWR model has the optimal fit. It may be the best model to explain the heterogeneity of the spatiotemporal distribution of LEED certified projects in the United States from 2017 to 2021. The GWR model and OLS model have a relatively poor fit. Notably, the TWR model fits better than the GWR model and is even very close to the GTWR model. This indicates that the temporal factor is more important than the spatial factor in the evolution of LEED certified project distribution in the United States from 2017 to 2021. Therefore, incorporating the temporal parameter into the geographically weighted model would be meaningful.

### 3.3 Results of influencing factor analysis

#### 3.3.1 Demographic, socioeconomic and environmental factors

**Fig. 4:**
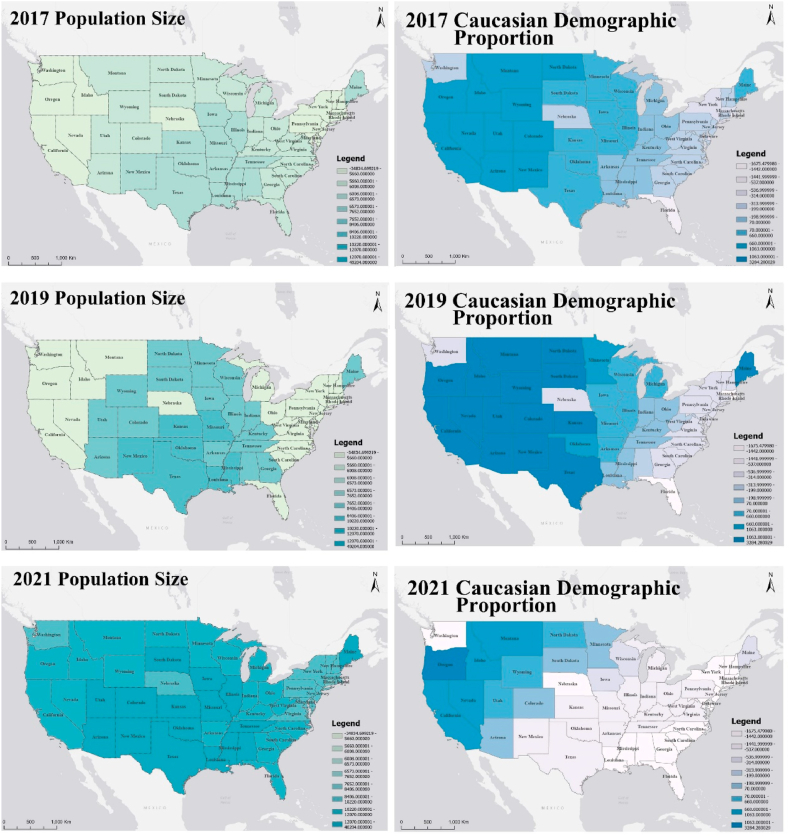
Coefficient distribution of influencing factors of population category.

**Fig. 5:**
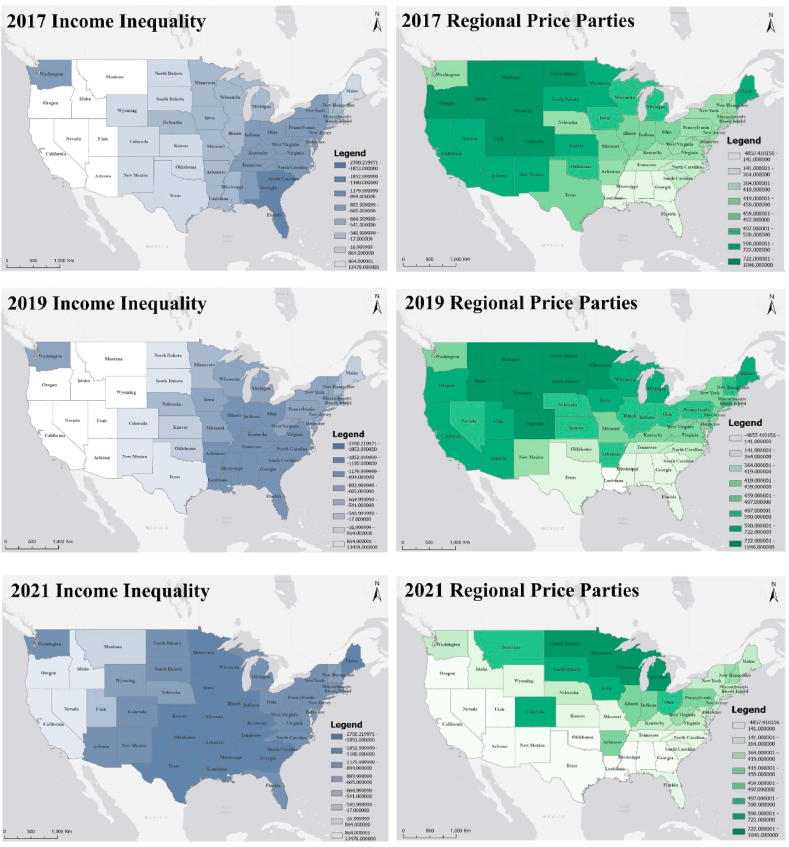
Coefficient distribution of influencing factors of socioeconomic category.

**Fig. 6:**
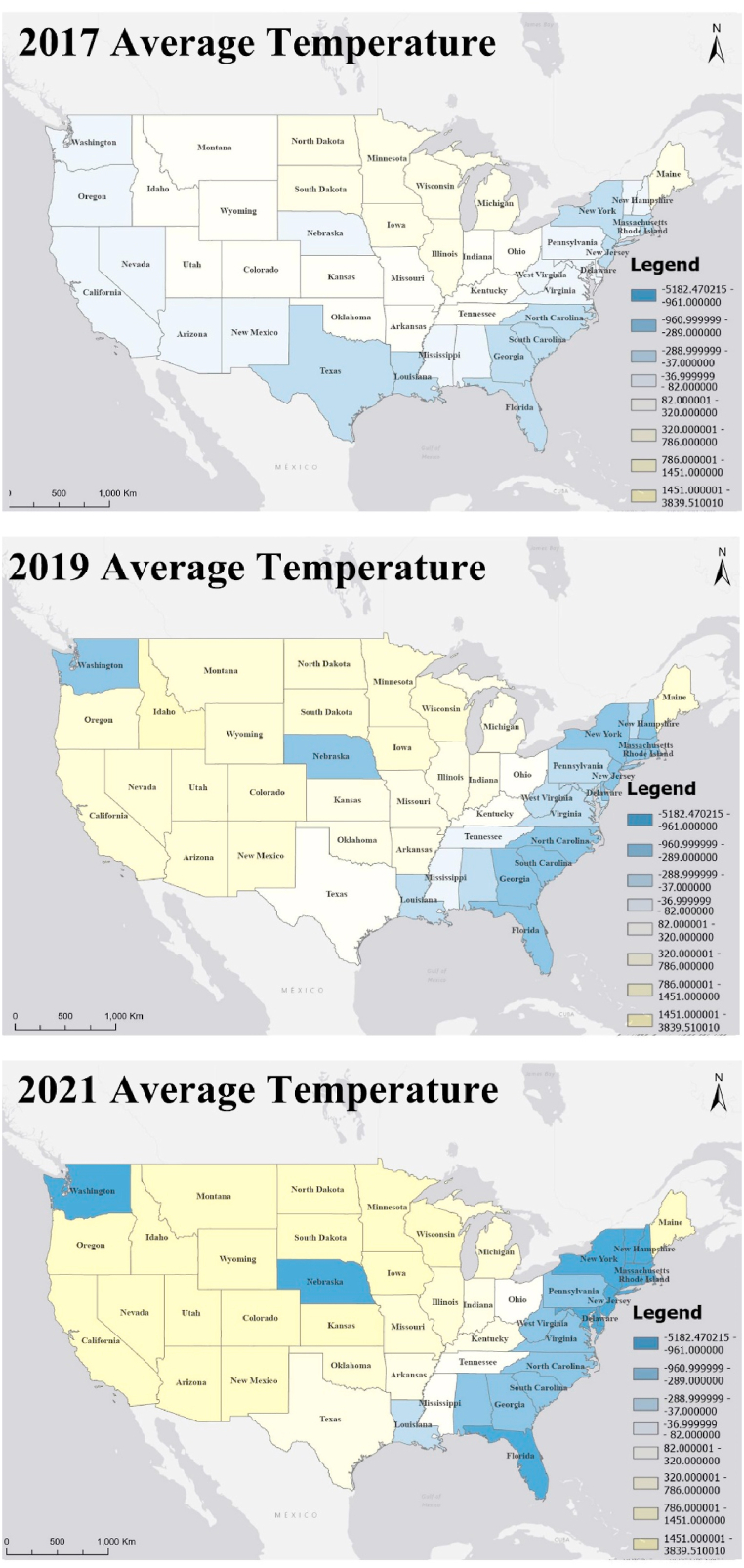
Coefficient distribution of influencing factors of environmental category.

The GTWR model not only has excellent goodness of fit but also provides trends in the spatiotemporal evolution of each influencing factor. Income inequality and the Caucasian demographic proportion in the total population are found to negatively influence the construction of LEED certified projects, while population size, regional price parities and the annual average temperature have a positive impact on it. The values of the GTWR statistical coefficients are given in Table 3. The mean and standard deviation of the regression coefficients for the population size (8236.1383 and 5164.9796, respectively) are higher than the other factors, ranging from −34834.7000 to 40204.0000, indicating that population size is the key parameter that has the most significant impact on the construction of LEED certified projects. It reveals that the higher the population, the higher the number of LEED certified projects constructed. Combined with Fig. 4, the strength of the correlation between population size and the implementation of LEED certified projects is increasing each year. The second most significant influencing factor is income inequality. The spatiotemporal distribution pattern is primarily positive for LEED certified project construction in the western region and negative in the eastern region, with the strength of the negative correlation expanding significantly, especially after 2021 (Fig. 5). The Caucasian demographic proportion and the construction of LEED certified projects show a pattern of positive correlation in the west and negative correlation in the east. The number of regions where regional price parity is positively correlated with the construction of LEED certified projects is decreasing and the strength of the correlation is diminishing. The strength of the effect of the annual average temperature on LEED certified projects is increasing year by year, roughly presenting a positive correlation in the west and central and a negative correlation pattern in Washington, Nebraska, and the eastern coast (Fig. 6).

**Table 3:** The coefficients value for the GTWR model.

Item	Intercept	Population Size	Caucasian Demographic Proportion	Income Inequality	Regional Price Parities	Average Temperature
Mean	713.0983	8236.1383	-18.9113	-533.1024	365.1813	227.1735
Min	-3523.4900	-34834.7000	-1675.4800	-2700.2200	-4857.4100	-5182.4700
Max	4374.0300	40204.0000	3284.2800	13478.9000	1046.0800	3839.5100
Sd	1938.9308	5164.9796	1041.41362	1529.2547	576.5823	1261.1424
25 %	-781.1185	6008.9875	-537.2870	-1180.4000	364.4610	-289.8155
50 %	1196.5850	7652.0300	-199.3250	-665.4295	459.8360	82.4716
75 %	2413.1326	10220.9502	660.5177	-17.9363	590.1825	786.3670

#### 3.3.2 Policymaking factors

To explore the relationship between regional differences in LEED certified project implementation and policymaking factors. This study used binary logistic regression to analyze the relationship between adopting different policy instruments in each state and the number of LEED certified projects. The results of binary logistic regression are shown in Supplementary Material Table S5. The results reveal that the adoption of three policy instruments - mandatory requirements, enabling or encouraging legislation, and density or high bonus - are statistically correlated with the increase in LEED certified projects (p-values less than 0.05), and particularly, the use of three policy instruments - expedited permitting, reduced fees, and property tax credit or exemption - are significantly correlated with the increase in LEED certified projects (p-values less than 0.01).

## 4 Discussion

### 4.1 Spatiotemporal difference distribution

Regional differences in the spatial distribution of LEED projects across building types may be due to the different green cost premiums between building types. Green-certified building costs are critical for the construction industry, especially compared to traditional buildings. Educational buildings have the highest average green cost premium of any type of building at 18 %. The green cost premium for office buildings is second at 6 %. The lowest green cost premium is found in residential buildings [40]. In terms of spatial distribution, by the end of 2021, residential buildings had the highest number of buildings (76.67 %), commercial buildings had the second highest number (14.34 %), followed by office buildings (4.51 %) and educational buildings (4.48 %). These types of green-certified buildings are more likely to be located in regions with higher levels of economic development, such as California, Illinois, Texas, and Maryland.

Significant regional differentiation is also exhibited between LEED projects with different certification levels. The majority of green buildings seek higher levels of LEED certification, often at or above the level of silver-certified [41]. And some studies have found that higher ratings have correspondingly higher costs for green-certified buildings. Silver-certified, gold-certified, and platinum-certified green buildings cost 0.23 %, 1.21 %, and 6.62 % more than LEED-certified, respectively [28]. The most significant number of LEED projects were silver-certified and gold-certified (36.32 % and 36.25 %, respectively, by the end of 2021). In recent years they are mostly found along the eastern and western coasts, in regions of higher economic development, such as California, New York, New Jersey, and Pennsylvania. Secondly, spatiotemporal hotspots for LEED-certified projects (15.17 %) are primarily located in Texas and Colorado, presumably because maintenance and repair costs for green-certified buildings with this rating are mostly less than others. Platinum-certified green buildings have the highest life cycle and construction costs. They are generally equipped with more technology and more expensive overall capital, maintenance, and operating expenses, so it has the lowest distribution in terms of numbers (12.27 %).

Factors that contribute to regional differentiation in the distribution of LEED projects across building types and certification levels may also include: (1) the maturity of the supply chain, especially for sustainable green-certified building products; (2) product-service competition; (3) the skills and work experience of project teams; (4) construction techniques and approaches; and (5) green-certified building policies and incentives.

### 4.2 Influential factors of spatiotemporal heterogeneity

#### 4.2.1 Demographic factors

As urbanization accelerates, governments with larger population sizes have formulated policies and implemented incentive programs to expand the supply of green certified buildings in response to a range of urban issues [42]. This study incorporates time effects and develops a spatiotemporal regression model, and the results also confirm that the number of LEED-certified projects is positively correlated with the size of the population, and that as a large number of people move to the city and the city's socioeconomics grows, LEED-certified projects are implemented more and more frequently [43]. And the strength of the correlation between population size and the implementation of LEED projects is increasing year by year and is gradually spreading to the east and west coasts. In addition, this study also focuses on demographic characteristics such as ethnicity, and finds that the Caucasian demographic proportion is mainly negatively correlated with the implementation of LEED certified projects. This indicates that regions with a high non-Caucasian demographic proportion distribute more LEED-certified projects. This may be in consideration of the potential shared health benefits of a vulnerable population, such as the population of non-Caucasian, to promote a more equitable and just development of LEED projects [14].

#### 4.2.2 Socioeconomic factors

It has been shown that economic capabilities are an important factor contributing to regional heterogeneity, and the government can promote the widespread implementation of green certified buildings by alleviating economic inequality and establishing a market-oriented development mechanism [12]. In this study, two indicators, income inequality and regional price parity, are used to measure the impact of socioeconomic factors on their distribution. In regions with higher income inequality, the distribution of LEED certified projects is often lower due to reduced social capital. One possible explanation is that regions with higher income inequality have a concentration of wealth in the hands of a minority, further decreasing the social capital to pursue a more extensive range of environmental projects. Wealthier or poorer regions are less likely to engage in sustainable development, which affects LEED project implementation. Regions with lower income inequality are less politically and socially homogeneous and therefore implement more LEED projects [44]. Regional price parity, reflecting consumer goods and services differences, helps compare living standards and well-being across regions [45]. The consumption price level tends to be close to GDP in expenditure [46]. Therefore, in regions with higher regional price parity, individuals appear to have a superior socioeconomic status that determines their willingness to pay for LEED projects [47].

#### 4.2.3 Environmental factors

For the study of building thermal environment, temperature is one of the most common parameters [48]. This study find that the average annual temperature is also tightly correlated with the spatiotemporal distribution patterns of LEED certified projects. The effects of climate change may have increased the intensity, duration, and frequency of extreme weather events such as heat waves and hailstorms [49]. As the understanding of lifestyle and environmental impacts gradually improves, there is a growing demand from homebuyers for buildings that can withstand any environmental changes and provide high-quality living conditions (e.g., LEED projects). Governments and real estate developers are also becoming more responsive to these demands [50]. As a result, LEED projects are increasingly favored for their capability to improve indoor environmental quality and environmental energy consumption.

#### 4.2.4 Policymaking factors

Numerous studies have confirmed that policy instruments adopted by the government are one of the most fundamental and effective ways to promote the implementation of green certified buildings [51]. This study investigated 569 incentive policies in the United States related to promoting the implementation of LEED projects and classified them into eight categories based on the policy instruments used in each policy. A binary logistic regression model was used to quantify the impact of these eight policy instruments on LEED project implementation. The results indicated that expedited permitting, reduced fees, and property tax credits or exemptions were found to be the most effective policy instruments and attractive incentives, especially in the early construction period.

Expedited permitting is a strategy that allows government authorities to offer significant incentives with little or no financial investment because such a strategy only requires a shift in permitting priorities [52,53]. Developers see this as an affordable and efficient way to reduce development costs. Because such incentives substantially shorten the complex and lengthy permitting process, decrease development time and funding, and diminish the soft costs related to the project (e.g., architect and legal fees, insurance premiums, and property taxes incurred during development). Similar to expedited permitting, density or height bonus requires little financial investment from municipalities. Many cities permit additional building density or height for LEED certified buildings. This is a desirable incentive, especially for developers in high-density cities.

Reducing fees is also a policy instrument that can encourage building stakeholders to pursue LEED projects. Some municipalities in the United States that charge fees for permit review or other permitting processes have begun to offer reductions or exemptions to developers who follow green-certified building standards. In many cases, such incentives are combined with expedited permitting. And this policy instrument does not directly affect the municipality's finances, as the proposed development generally increases the assessed property value of the city so that the municipality will not be threatened with a loss of revenue.

Property tax credit or exemption is also regarded as a preferable incentive for LEED projects. This policy instrument involves no financial expense for local authorities to implement and has the advantage of being a post facto incentive, enabling investors to receive monetary compensation after constructing a LEED certified project. This type of tax incentive is effective, especially when the investment budget is high [54]. Therefore, providing property tax credit or exemption for green-certified buildings can stimulate and encourage the growth of LEED projects.

In addition, mandatory requirements are the most popular policy instrument for government authorities, followed by enabling or encouraging legislation and density or high bonus (see Supplementary Material Table S6, Table S7, and Fig. S5 for more details).

### 4.3 Implications

In the construction industry, the green building mode is still in the development stage in most regions of the world, and research on it is still limited [55]. Therefore, investigating the spatiotemporal distribution characteristics of experienced United States LEED certified projects and their influencing factors can accelerate green renewal and stimulate the development of green-certified buildings in regions with lower green-certified building development. And the successful experience of LEED certified projects in the United States can also guide the next stage of ultra-low energy buildings, near-zero energy buildings, and zero energy buildings [56]. In addition, many low carbon and green technologies, such as green roofs, green walls, photovoltaic panels, and heat pumps, are not always distributed equitably in cities. Their unequal accessibility is increasingly seen as an environmental justice issue [37,[57], [58], [59]]. Therefore, understanding the spatiotemporal distribution experience of LEED certified projects in the United States can contribute to the optimal siting of low carbon and green technologies, promoting their more equitable and widespread distribution in cities.

This study investigates the influencing factors of the spatiotemporal distribution of green buildings, examining the interaction between controllable and uncontrollable factors. Focusing solely on controllable factors could lead to overlooking the diversity and complexity of the real world. Despite their lack of direct intervention potential, uncontrollable factors play a crucial role in practical contexts. Factors like population size, Caucasian demographic proportion, income inequality, and average temperature, which are intrinsic characteristics of cities and communities, significantly impact the building market and housing demand. For instance, increasing population size could elevate energy consumption and environmental stress [60]. Average temperature affects energy efficiency, material selection, and indoor comfort of green-certified buildings [61]. Caucasian demographic proportion and income inequality may reflect societal structure and resource allocation, influencing the demand and selection of green-certified buildings [62]. Investigating these uncontrollable factors contributes to a more comprehensive understanding of the underlying dynamic mechanisms driving the adoption of green-certified buildings, thereby identifying potential opportunities and challenges. Furthermore, this study deeply investigates controllable factors such as regional price parity and policy instruments, which can be adjusted through government policies and market interventions. For example, implementing incentives, rewards, high-level green building standards, financing mechanisms, streamlined approval processes, strengthened policy supervision and enforcement, fostering regional collaboration, and establishing demonstration and pilot projects for green-certified buildings, as well as adjusting regional pricing, can facilitate the widespread implementation of green-certified buildings [[63], [64], [65], [66]]. Hence, delving into the intricate relationships between controllable and uncontrollable factors is crucial for advancing the extensive adoption of green-certified buildings.

The following recommendations (Table 4) are summarized based on experience gained from LEED certified projects in the United States.

**Table 4:** Policy suggestions and their applicable regions.

Policy Suggestions	Applicable Regions
Widely implement green-certified buildings	Large population, high regional price parity and frequent extreme weather events
Fairly implement green-certified buildings	Large non-Caucasian population and serious income inequality
Use policy instruments such as Requirement, Enabling or Encouraging Legislation, Reduced Fees and Density or Height Bonus	Large population, high international migration rate, serious income inequality and high regional price parity
Use policy instruments such as Expedited Permitting and Marketing or Technical Assistance	Large population, high international migration rate, high annual average temperature, and many heating and cooling days
Use the policy instrument of Property Tax Credit or Exemption	Higher educational attainment
Use the policy instrument of Financing	Large population

### 4.4 Limitations

Firstly, the study was conducted at the state level due to data availability. Empirical analysis at the state level using the GTWR revealed patterns in the spatiotemporal heterogeneity of LEED certified projects and influencing factors in the United States. Nevertheless, the growth in green-certified building implementation has been dramatic in recent years, and a larger sample of green-certified building studies will emerge. Further analysis at a smaller spatial scale is possible in future studies.

Second, this study explored the pattern of spatiotemporal heterogeneity of LEED certified projects in the United States without dividing green-certified buildings by building type. Because different types of green-certified buildings have different occupancy rights, they may be driven by different factors. It is therefore recommended that in future research, it may be worthwhile to categorize green-certified buildings by building type and then explore their influencing factors.

Finally, this study used an ordinary binary logistic regression to analyze incentives for LEED projects in the United States. It is recommended that more advanced analytical methods (e.g., tripartite evolutionary game theory analysis, synergistic analysis, data envelopment analysis, and social network analysis) be used in the following study to further explore the effectiveness of various policy instruments in greater depth to assist the government in formulating more efficacious incentive policies.

## 5 Conclusion

This study conducted a spatiotemporal analysis of LEED certified projects in the United States with the state-level panel dataset from 2000 to 2021. Genetic algorithms and space-time cube analysis explored their spatiotemporal distribution patterns. Temporal and spatial non-stationarity were incorporated into a weighted model to explain the spatiotemporal heterogeneity of LEED certified projects. The evolutionary trends of five influencing factors were explored based on GTWR model results. In addition, the policies that incentivize the implementation of LEED certified programs in the United States were also reviewed, and the impact of policymaking factors on LEED certified project implementation was analyzed using binary logistic regression.

The results show that: (1) significant regional differences and clustering exist in the distribution of LEED projects of different building types and certification levels; (2) The influencing factors contributing to the spatiotemporal heterogeneity pattern of LEED certified projects are population size, the Caucasian demographic proportion to the total population, income inequality, regional price parity, and average annual temperature (the mean values of the coefficients for each factor were 8236.1383, −18.9113, −533.1024, 365.1813 and 227.1735 respectively); (3) Policymaking factors may also influence the distribution of LEED certified projects, particularly through using three policy instruments: expedited permitting, reduced fees, and property tax credit or exemption (p-values less than 0.01).

Findings from this study will help policymakers be better informed about green-certified building implementation and assist them in formulating and implementing more efficient and appropriate incentive policies to promote more green-certified buildings. It will also guide the next stage of spatial siting of ultra-low energy buildings, near-zero energy buildings, and zero energy buildings, and promote more equitable distribution of low carbon and green technologies.

## CRediT authorship contribution statement

**Siwei Chen:** Data curation, Formal analysis, Investigation, Methodology, Writing – original draft. **Zhonghua Gou:** Conceptualization, Project administration, Supervision, Writing – review & editing.

## Declaration of competing interest

The authors declare that they have no known competing financial interests or personal relationships that could have appeared to influence the work reported in this paper.
