# Supplementary material for: Spatiotemporal distribution of green-certified buildings and the influencing factors: A study of U.S
Source: Heliyon. 2023 Oct 31;9(11):e21868. doi: 10.1016/j.heliyon.2023.e21868 (PMC10660489; doi:10.1016/j.heliyon.2023.e21868)
Supplement: Multimedia component 1 [file mmc1.docx]

**Supplementary Material**

**Table S1.** Description of the Number of LEED Projects in the Fifty States of the United States.

| **Location** | **Certification Year** | | | **Certification level** | | | | **Building type** | | | |
| --- | --- | --- | --- | --- | --- | --- | --- | --- | --- | --- | --- |
|  | **2017** | **2019** | **2021** | **LEED** | **Silver** | **Gold** | **Platinum** | **Residential** | **Commercial** | **Office** | **Educational** |
| Alabama | 177 | 187 | 201 | 58 | 79 | 57 | 7 | 31 | 26 | 5 | 2 |
| Alaska | 242 | 250 | 261 | 19 | 139 | 101 | 2 | 67 | 2 | 4 | 0 |
| Arizona | 980 | 1392 | 2507 | 152 | 1069 | 1066 | 220 | 1707 | 116 | 14 | 6 |
| Arkansas | 391 | 416 | 432 | 256 | 97 | 65 | 14 | 229 | 62 | 8 | 8 |
| California | 6328 | 7606 | 8591 | 1466 | 2129 | 3199 | 1797 | 2493 | 1440 | 121 | 67 |
| Colorado | 1271 | 1861 | 2299 | 818 | 550 | 732 | 199 | 1111 | 195 | 26 | 35 |
| Connecticut | 469 | 512 | 569 | 115 | 107 | 274 | 73 | 262 | 68 | 4 | 18 |
| Delaware | 52 | 63 | 64 | 22 | 25 | 13 | 4 | 5 | 11 | 0 | 3 |
| Florida | 2308 | 2658 | 2818 | 740 | 815 | 940 | 323 | 1050 | 421 | 52 | 52 |
| Georgia | 1018 | 1104 | 1177 | 182 | 416 | 523 | 56 | 234 | 183 | 24 | 26 |
| Hawaii | 951 | 1029 | 1087 | 42 | 752 | 180 | 113 | 818 | 20 | 7 | 6 |
| Idaho | 304 | 380 | 425 | 43 | 100 | 134 | 148 | 228 | 27 | 7 | 3 |
| Illinois | 1494 | 1847 | 2005 | 393 | 826 | 630 | 156 | 311 | 406 | 20 | 92 |
| Indiana | 632 | 715 | 760 | 117 | 392 | 218 | 33 | 241 | 61 | 5 | 12 |
| Iowa | 270 | 301 | 307 | 91 | 98 | 101 | 17 | 10 | 56 | 6 | 0 |
| Kansas | 170 | 191 | 211 | 44 | 95 | 53 | 19 | 247 | 96 | 11 | 12 |
| Kentucky | 230 | 266 | 302 | 79 | 83 | 131 | 9 | 75 | 28 | 16 | 7 |
| Louisiana | 602 | 608 | 661 | 138 | 377 | 54 | 92 | 496 | 17 | 3 | 15 |
| Maine | 145 | 154 | 171 | 36 | 61 | 51 | 23 | 8 | 9 | 8 | 1 |
| Maryland | 1191 | 1555 | 2517 | 562 | 1003 | 867 | 85 | 665 | 191 | 17 | 92 |
| Massachusetts | 1206 | 1440 | 1597 | 207 | 425 | 758 | 207 | 199 | 340 | 13 | 46 |
| Michigan | 1184 | 1324 | 1408 | 266 | 527 | 528 | 87 | 639 | 113 | 14 | 16 |
| Minnesota | 434 | 477 | 525 | 160 | 152 | 151 | 62 | 58 | 85 | 7 | 7 |
| Mississippi | 111 | 119 | 124 | 38 | 27 | 55 | 4 | 30 | 13 | 4 | 1 |
| Missouri | 496 | 553 | 583 | 124 | 156 | 140 | 163 | 151 | 87 | 11 | 13 |
| Montana | 107 | 118 | 128 | 41 | 29 | 39 | 19 | 11 | 13 | 10 | 1 |
| Nebraska | 78 | 84 | 86 | 31 | 27 | 23 | 5 | 2 | 19 | 5 | 3 |
| Nevada | 367 | 404 | 427 | 33 | 224 | 102 | 68 | 191 | 25 | 5 | 0 |
| New Hampshire | 149 | 157 | 164 | 32 | 58 | 66 | 8 | 20 | 9 | 2 | 0 |
| New Jersey | 1208 | 1394 | 1514 | 222 | 486 | 667 | 139 | 567 | 114 | 8 | 13 |
| New Mexico | 1576 | 1800 | 2044 | 58 | 475 | 390 | 1121 | 1397 | 32 | 12 | 64 |
| New York | 2300 | 2890 | 3253 | 484 | 1352 | 1209 | 208 | 526 | 666 | 10 | 19 |
| North Carolina | 1621 | 1761 | 1836 | 290 | 936 | 541 | 69 | 837 | 214 | 19 | 28 |
| North Dakota | 47 | 57 | 59 | 26 | 19 | 12 | 2 | 5 | 7 | 2 | 0 |
| Ohio | 2065 | 2621 | 3060 | 493 | 1356 | 1018 | 193 | 1481 | 202 | 23 | 259 |
| Oklahoma | 138 | 155 | 163 | 43 | 76 | 44 | 0 | 37 | 32 | 4 | 4 |
| Oregon | 992 | 1078 | 1139 | 278 | 182 | 464 | 215 | 508 | 125 | 10 | 17 |
| Pennsylvania | 1763 | 2049 | 2249 | 564 | 707 | 825 | 153 | 792 | 237 | 27 | 49 |
| Rhode Island | 92 | 102 | 112 | 19 | 31 | 59 | 3 | 3 | 11 | 1 | 0 |
| South Carolina | 432 | 476 | 494 | 154 | 213 | 110 | 17 | 119 | 39 | 10 | 15 |
| South Dakota | 72 | 82 | 94 | 24 | 47 | 19 | 4 | 2 | 10 | 3 | 1 |
| Tennessee | 617 | 674 | 720 | 125 | 297 | 227 | 71 | 235 | 103 | 13 | 22 |
| Texas | 5227 | 7804 | 10968 | 8562 | 1202 | 1062 | 142 | 6408 | 384 | 55 | 80 |
| Utah | 297 | 354 | 387 | 81 | 141 | 123 | 42 | 22 | 43 | 21 | 4 |
| Vermont | 98 | 104 | 108 | 22 | 35 | 38 | 13 | 8 | 14 | 0 | 0 |
| Virginia | 1491 | 1805 | 1981 | 777 | 632 | 515 | 57 | 549 | 275 | 24 | 34 |
| Washington | 1219 | 1433 | 1573 | 231 | 460 | 619 | 263 | 373 | 215 | 37 | 7 |
| West Virginia | 28 | 39 | 41 | 15 | 14 | 7 | 5 | 0 | 8 | 0 | 2 |
| Wisconsin | 415 | 455 | 478 | 126 | 152 | 158 | 42 | 20 | 88 | 19 | 4 |
| Wyoming | 105 | 108 | 111 | 11 | 40 | 49 | 11 | 39 | 11 | 1 | 0 |

**
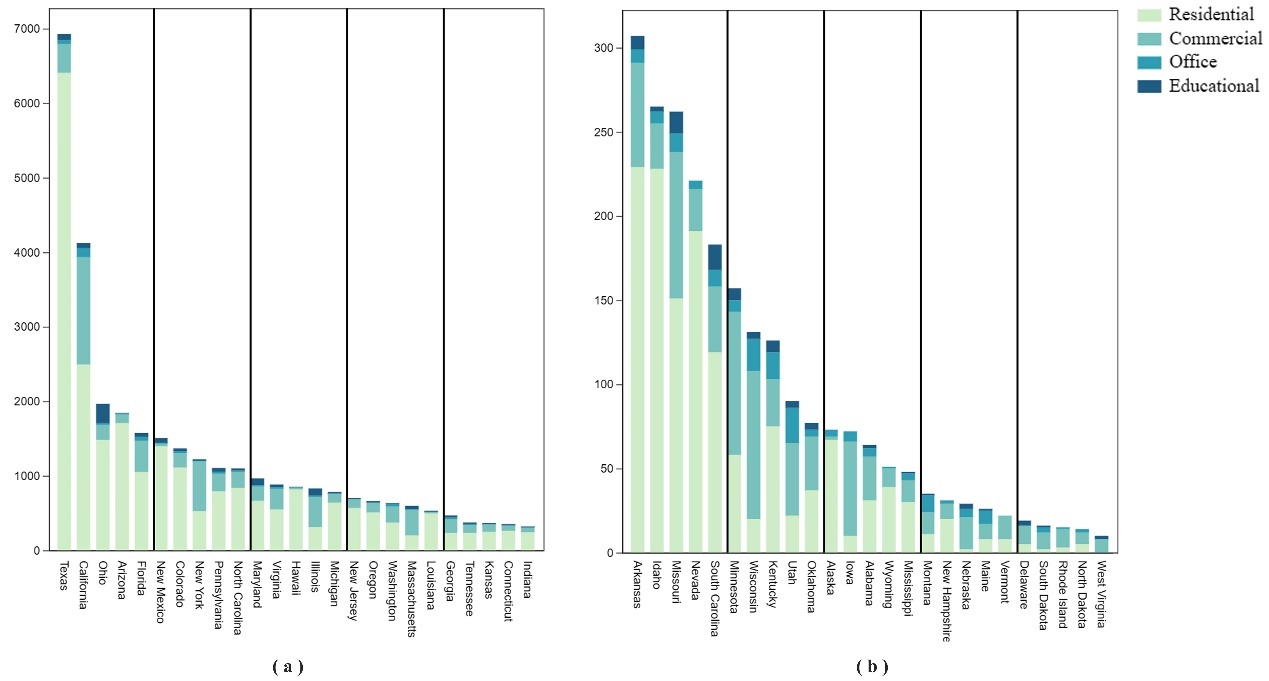
**

**Fig. S1.** Number of LEED projects of different building types per state. (a) top 25 states with higher numbers; (b) bottom 25 states with lower numbers.

**
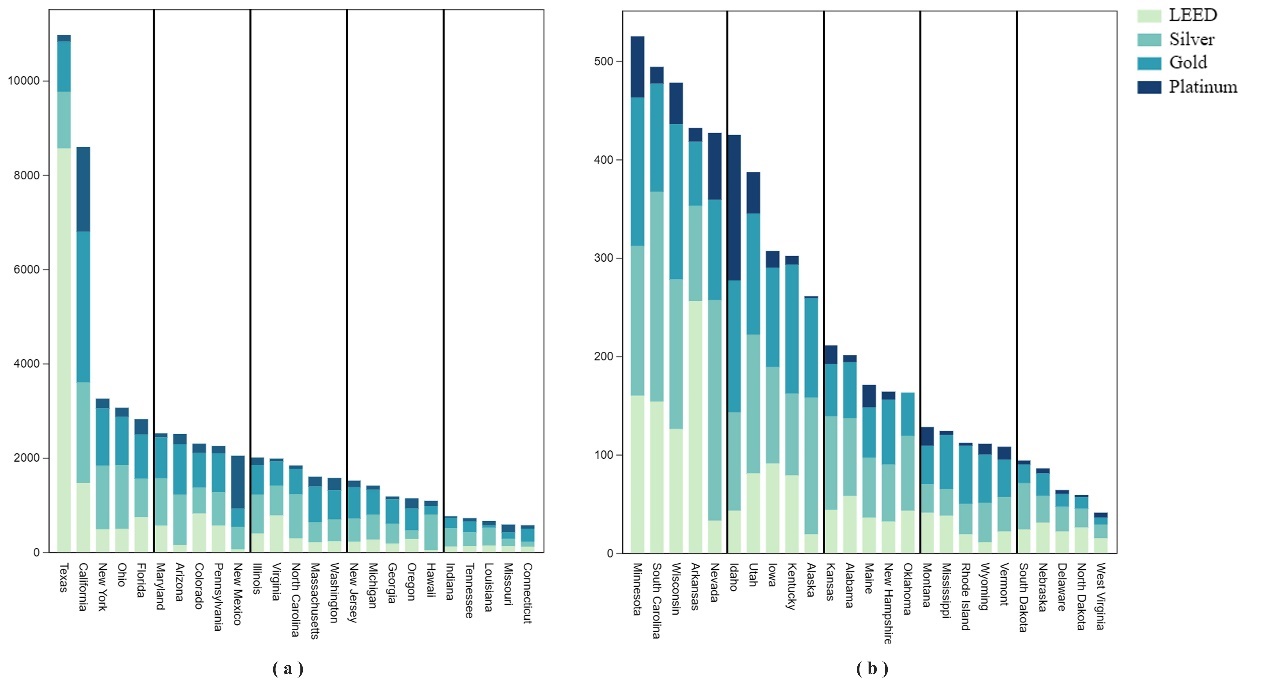
**

**Fig. S2.** Number of LEED projects at different certification levels per state. (a) top 25 states with higher numbers; (b) bottom 25 states with lower numbers.

**Table S2.** Description of potential explanatory variables and data sources.

| **Category** | **Explanatory variable** | **Description** | **Source** | **Reference** |
| --- | --- | --- | --- | --- |
| Demographic | Population size | Population size within a state | National Historical Geographic Information System | (Gao et al., 2020) |
|  | Population density | Population size/Area of the state | National Historical Geographic Information System | (Gao et al., 2020) |
|  | population ages 65 and above | Population 65 years of age or older as a percentage of the total population | National Historical Geographic Information System | (Zou et al., 2017) |
|  | Caucasian demographic proportion | Caucasian population as a percentage of the total population | National Historical Geographic Information System | (Yan, Hui et al., 2022; Zou et al., 2017) |
|  | African American demographic proportion | African American population as a percentage of the total population | National Historical Geographic Information System | (Yan, Hui et al., 2022; Zou et al., 2017) |
|  | Indigenous demographic proportion | American Indian and Alaska Native population as a percentage of the total population | National Historical Geographic Information System | (Yan, Hui et al., 2022; Zou et al., 2017) |
|  | Asian demographic proportion | Asian population as a percentage of the total population | National Historical Geographic Information System | (Yan, Hui et al., 2022; Zou et al., 2017) |
|  | Pacific Islander demographic proportion | Native Hawaiian and other Pacific Islander population as a percentage of the total population | National Historical Geographic Information System | (Yan, Hui et al., 2022; Zou et al., 2017) |
|  | International migration rate | population of people moving across the border for the purpose of settling in other countries as a percentage of the total population | National Historical Geographic Information System | (Han et al., 2023) |
|  | Educational attainment | Public college graduation rate for people ages 25 and above | World population review | (Lee and Koski, 2012) |
|  | Life expectancy | Average number of years that a newborn could expect to live | World population review | (Han et al., 2023) |
|  | Mortality rate infant | The ratio of the number of deaths under the age of one year after birth to the number of births | Centers for disease control and prevent | (Han et al., 2023) |
| Socioeconomic | GDP per capita | Gross Domestic Product per capita | National Historical Geographic Information System | (Lee and Koski, 2012) |
|  | Median Household Income | Measure the income level of general public in a state | National Historical Geographic Information System | (Lee and Koski, 2012) |
|  | Income inequality | Measure the inequality of income distribution across a population | National Historical Geographic Information System | (Zou et al., 2017) |
|  | Regional price parities | Measure the differences in price levels across states within a year. | U.S. Bureau of Economic Analysis | (Yan, Hui et al., 2022; Zou et al., 2017) |
|  | Unemployment rate | The share of the labor force that is without work but available for employment. | National Historical Geographic Information System | (Yan et al., 2022; Zou et al., 2017) |
|  | Human development index | Composite indicator of education, health and living standards | United Nations Development Programme | (Han et al., 2023) |
| Environmental | Annual precipitation | Annual average | National Centers for Environmental Information | (Han et al., 2023) |
|  | Annual average temperature | Annual average | National Centers for Environmental Information | (Lee and Koski, 2012) |
|  | Cooling degree days | The accumulated degree of daily average temperature higher than 65 ° F (18.3 ° C) in a year | National Centers for Environmental Information | (Lee and Koski, 2012) |
|  | Heating degree days | The accumulated degree of daily average temperature below 65 ° F (18.3 ° C) in a year | National Centers for Environmental Information | (Lee and Koski, 2012) |
|  | Mean elevation | Mean elevation | Maps and Statistics of the World and Regions | (Han et al., 2023) |
|  | Distance to the nearest coastline | Distance from a state to the nearest coastline | ArcGIS Online | (Chen et al., 2022) |

**Table S3.** Summary statistics of explanatory variables.

| **Explanatory Variables** | **Min** | **Max** | **Mean** | **SD** |
| --- | --- | --- | --- | --- |
| Population size | 457128 | 39283497 | 6052375.77 | 6813014.894 |
| Caucasian demographic proportion | 23.6873 | 94.6442 | 75.576773 | 12.9196199 |
| Income inequality | 0.418 | 0.5142 | 0.464207 | 0.0183316 |
| Regional price parities | 55.417 | 164.896 | 91.80641 | 25.53894 |
| Annual average temperature | 26.5 | 73.2 | 52.966 | 8.6849 |


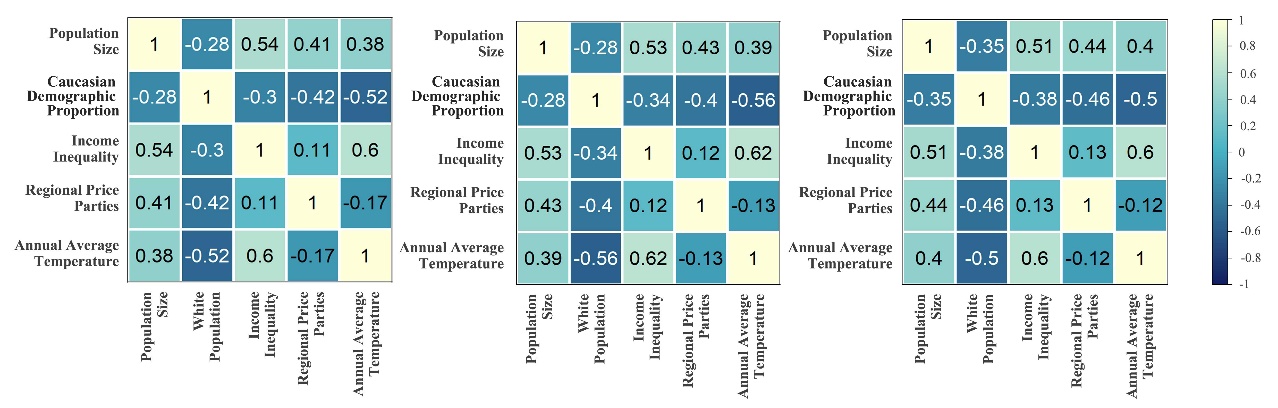


(a) （b） （c）

**Fig. S3.** Correlation coefficient between variables. (a) 2017; (b) 2019; (c) 2021.

More details of the analytical methods mentioned in the methodological of this study are as follows.

Global spatial autocorrelation is represented by equation (1).

$I_{G}=\frac{n\Sigma_{ⅈ=1}^{n}\Sigma_{j=1}^{n}w_{ij}\left( x_{i}-\bar{x} \right)\left( x_{j}-\bar{x} \right)}{\Sigma_{ⅈ=1}^{n}\Sigma_{j=1}^{n}w_{ij}\left( x_{i}-\bar{x} \right)^{2}}$ (1)

$I_{G}$ represents global Moran’s Index; n is the total number of spatial cells; $x_{i}$ and

$x_{j}$ represent the number of LEED certified projects in region i and region j; $\bar{x}$ represents the average value of LEED certified projects；$w_{ij}$ is an element in the spatial weight coefficient of regions i and j, which is used to indicate the adjacency of spatial regions i and j. Global Moran's Index ranges from -1 to 1 , where a value higher than 0 indicates a positive spatial correlation with a clustering tendency, while a value lower than 0 indicates a negative spatial correlation with a dispersion tendency, and when the value is approximately equal to 0 indicates a random distribution.

Local spatial autocorrelation is represented by equation (2).

$I_{L}=\frac{\left( x_{i}-\bar{x} \right)}{S^{2}}\Sigma_{j\neq i}w_{ij}\left( x_{j}-\bar{x} \right)$ （2）

Here，$s^{2}=\frac{\sum_{i=1}^{n} \left( x_{i}-\bar{x} \right)}{n}$. $I_{L}$ represents Local Moran’s Index. The rest of the explanation is the same as Global Moran’s Index above. Local Moran’s Index identifies spatial clustering and outliers in LEED certified projects. A value of Local Moran’s Index higher than 0 means that the local spatial cells are spatially positively correlated with adjacent spatial cells, representing a "high-high" cluster or "low-low" cluster; a value of Local Moran's Index lower than 0 means that the local spatial cells are spatially negatively correlated with adjacent spatial cells, represented as a "low-high" outlier or a "high-low" outlier.

Ordinary least squares (OLS) regression is represented by equation (3).

$y_{i}=\beta_{o}+\sum_{k=1}^{p} \beta_{k}x_{ik}+\varepsilon_{i}$ （3）

$y_{i}$ is the value at location i；$x_{ik}$ represents the kth independent variable at location i；$\beta_{o}$ is the intercept at location i；$\beta_{k}$ is the local coefficient at location i；p is the number of factors；$\varepsilon_{i}$ represents the random error term.

Geographically weighted regression (GWR)is represented by equation (4).

$y_{i}=\beta_{o}\left( u_{i},\nu_{i} \right)+\Sigma_{k=1}^{p}\beta_{k}\left( u_{i},\nu_{i} \right)x_{ik}+\varepsilon_{i}$ （4）

$y_{i}$ is the value of location i；$\left( u_{i},\nu_{i} \right)$ is the geographic coordinate of location i；$\beta_{o}\left( u_{i},\nu_{i} \right)$ and $\beta_{k}\left( u_{i},\nu_{i} \right)$ are the intercept and local regression coefficient at location i；p is the number of factors；$x_{ik}$ is the k-th independent variable at location i；$\varepsilon_{i}$ is the random error term at location i.

Temporally weighted regression (TWR) is represented by equation (5).

$y_{i}=\beta_{o}\left( t \right)+\sum_{k=1}^{p} \beta_{k}\left( t \right)x_{ik}+\varepsilon_{i}$ （5）

$y_{i}$ is the dependent variable at time t；$x_{ik}$ is the k-th independent variable at time t；p is the number of independent variables； $\beta_{o}$ is the intercept at time t；$\beta_{k}$ is the local regression coefficient of the k-th independent variable at time t；$\varepsilon_{i}$ is the random error term at time t.

Geographically and temporally weighted regression (GTWR) is represented by equation (6).

$y_{i}=\beta_{0}\left( u_{i},\nu_{i_{1}}t_{i} \right)+\sum_{k=1}^{p} \beta_{k}\left( u_{i}\nu_{i},t_{i} \right)x_{ik}+\varepsilon_{i}$ （6）

$y_{i}$ is the dependent variable at time t；$x_{ik}$ is the k-th independent variable at time t；p is the number of independent variables；since there are two variables in the model, m = 2; $\beta_{o}$ is the intercept at time t；$\beta_{k}$ is the local regression coefficient of the k-th independent variable at time t；$\varepsilon_{i}$ is the random error term at time t；$u_{i}$ and $\nu_{i}$ represent the geographic position of location I; $t_{i}$ represents the time position of location i.

**Table S4.** Global Moran’s Index of LEED projects in the United States from 2017 to 2021.

| **Year** | **2017** | **2019** | **2021** |
| --- | --- | --- | --- |
| **Global Moran's Index** | 0.017202 | 0.03274 | 0.062217 |
| **z-score** | 2.371065 | 4.499598 | 8.515022 |
| **p-value** | 0.017737 | 0.000007 | 0.000000 |


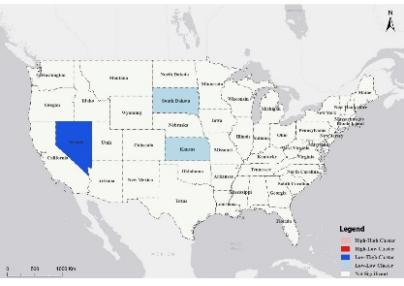

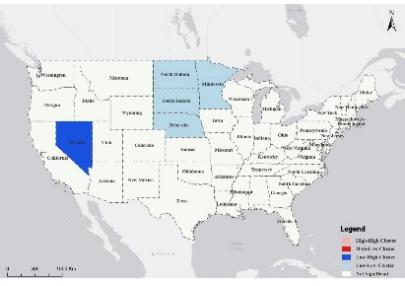

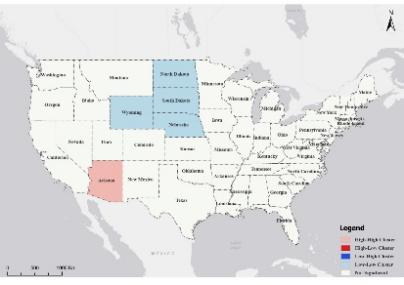


**Fig. S4.** Local spatial correlation clusters. (a) 2017; (b) 2019; (c) 2021.

**Table S5.** Results of binary logistic regression model.

| **Variable** | **p** | **Odds Ratio** | **95% CI for Odds Ratio** | |
| --- | --- | --- | --- | --- |
|  |  |  | **Lower** | **Upper** |
| Requirement | 0.038 | 8.522 | 1.127 | 64.447 |
| Enabling/Encouraging Legislation | 0.027 | 2.332 | 1.103 | 4.927 |
| Expedited Permitting | 0.009 | 3.477 | 1.374 | 8.800 |
| Reduced Fees | 0.002 | 3.927 | 1.650 | 9.348 |
| Density/Height Bonus | 0.012 | 2.684 | 1.244 | 5.789 |
| Property Tax Credit / Exemption | 0.005 | 3.459 | 1.469 | 8.145 |
| Marketing/Technical Assistance | 0.160 | 1.981 | 0.763 | 5.148 |
| Financing | 0.079 | 2.347 | 0.907 | 6.074 |

**Table S6.** Number of policies including Requirement, Enabling or Encouraging Legislation, Expedited Permitting and Reduced Fees.

| **State** | **Policy Instrument** | | | |
| --- | --- | --- | --- | --- |
|  | Requirement | Enabling or Encouraging Legislation | Expedited Permitting | Reduced Fees |
| Alabama | 0 | 0 | 0 | 0 |
| Alaska | 2 | 1 | 0 | 1 |
| Arizona | 9 | 0 | 2 | 1 |
| Arkansas | 1 | 1 | 0 | 0 |
| California | 73 | 11 | 13 | 5 |
| Colorado | 12 | 1 | 2 | 1 |
| Connecticut | 3 | 0 | 0 | 1 |
| Delaware | 1 | 0 | 1 | 0 |
| Florida | 26 | 5 | 21 | 15 |
| Georgia | 11 | 2 | 0 | 0 |
| Hawaii | 2 | 0 | 0 | 0 |
| Idaho | 5 | 0 | 0 | 0 |
| Illinois | 13 | 7 | 3 | 5 |
| Indiana | 2 | 0 | 0 | 0 |
| Iowa | 0 | 0 | 0 | 0 |
| Kansas | 1 | 0 | 0 | 0 |
| Kentucky | 1 | 3 | 1 | 2 |
| Louisiana | 1 | 0 | 0 | 0 |
| Maine | 5 | 1 | 0 | 0 |
| Maryland | 14 | 1 | 1 | 0 |
| Massachusetts | 11 | 2 | 0 | 1 |
| Michigan | 3 | 5 | 0 | 0 |
| Minnesota | 4 | 2 | 0 | 1 |
| Mississippi | 1 | 1 | 0 | 0 |
| Missouri | 6 | 2 | 0 | 0 |
| Montana | 1 | 0 | 0 | 0 |
| Nebraska | 0 | 0 | 0 | 0 |
| Nevada | 6 | 1 | 0 | 1 |
| New Hampshire | 1 | 0 | 0 | 1 |
| New Jersey | 10 | 12 | 1 | 1 |
| New Mexico | 4 | 0 | 1 | 0 |
| New York | 17 | 6 | 0 | 5 |
| North Carolina | 6 | 0 | 0 | 5 |
| North Dakota | 0 | 0 | 0 | 0 |
| Ohio | 4 | 3 | 0 | 3 |
| Oklahoma | 1 | 1 | 0 | 0 |
| Oregon | 4 | 1 | 1 | 0 |
| Pennsylvania | 6 | 0 | 0 | 2 |
| Rhode Island | 2 | 1 | 0 | 0 |
| South Carolina | 3 | 0 | 1 | 0 |
| South Dakota | 1 | 0 | 0 | 0 |
| Tennessee | 4 | 0 | 0 | 1 |
| Texas | 8 | 1 | 0 | 0 |
| Utah | 3 | 1 | 1 | 1 |
| Vermont | 0 | 0 | 0 | 0 |
| Virginia | 7 | 3 | 0 | 0 |
| Washington | 12 | 3 | 4 | 1 |
| West Virginia | 1 | 0 | 0 | 0 |
| Wisconsin | 2 | 1 | 0 | 0 |
| Wyoming | 0 | 0 | 0 | 0 |
| **Sum** | 310 | 79 | 53 | 54 |

**Table S7.** Number of policies including Density or Height Bonus, Property Tax Credit or Exemption, Marketing or Technical Assistance and Financing.

| **State** | **Policy Instrument** | | | |
| --- | --- | --- | --- | --- |
|  | Density or Height Bonus | Property Tax Credit or Exemption | Marketing or Technical Assistance | Financing |
| Alabama | 0 | 0 | 0 | 0 |
| Alaska | 0 | 0 | 0 | 0 |
| Arizona | 0 | 1 | 0 | 0 |
| Arkansas | 0 | 0 | 0 | 0 |
| California | 6 | 1 | 4 | 1 |
| Colorado | 0 | 0 | 0 | 0 |
| Connecticut | 1 | 1 | 0 | 0 |
| Delaware | 0 | 0 | 0 | 0 |
| Florida | 12 | 0 | 7 | 2 |
| Georgia | 3 | 1 | 0 | 0 |
| Hawaii | 0 | 0 | 0 | 0 |
| Idaho | 2 | 0 | 0 | 0 |
| Illinois | 1 | 0 | 0 | 2 |
| Indiana | 0 | 3 | 0 | 0 |
| Iowa | 1 | 1 | 0 | 0 |
| Kansas | 0 | 1 | 0 | 0 |
| Kentucky | 1 | 0 | 1 | 1 |
| Louisiana | 1 | 0 | 1 | 0 |
| Maine | 1 | 0 | 0 | 0 |
| Maryland | 0 | 9 | 0 | 0 |
| Massachusetts | 1 | 0 | 0 | 0 |
| Michigan | 2 | 0 | 0 | 1 |
| Minnesota | 1 | 0 | 0 | 0 |
| Mississippi | 0 | 0 | 0 | 0 |
| Missouri | 0 | 0 | 0 | 0 |
| Montana | 0 | 0 | 0 | 0 |
| Nebraska | 0 | 0 | 0 | 0 |
| Nevada | 3 | 2 | 1 | 0 |
| New Hampshire | 1 | 0 | 0 | 0 |
| New Jersey | 4 | 0 | 0 | 0 |
| New Mexico | 0 | 1 | 0 | 0 |
| New York | 9 | 6 | 0 | 1 |
| North Carolina | 1 | 1 | 0 | 0 |
| North Dakota | 0 | 0 | 0 | 0 |
| Ohio | 3 | 3 | 0 | 0 |
| Oklahoma | 0 | 0 | 0 | 0 |
| Oregon | 1 | 2 | 1 | 2 |
| Pennsylvania | 10 | 0 | 0 | 1 |
| Rhode Island | 0 | 1 | 0 | 0 |
| South Carolina | 1 | 0 | 1 | 1 |
| South Dakota | 0 | 0 | 0 | 0 |
| Tennessee | 2 | 0 | 0 | 1 |
| Texas | 0 | 2 | 0 | 0 |
| Utah | 0 | 0 | 0 | 0 |
| Vermont | 1 | 0 | 0 | 0 |
| Virginia | 1 | 3 | 1 | 0 |
| Washington | 6 | 2 | 2 | 0 |
| West Virginia | 0 | 0 | 0 | 0 |
| Wisconsin | 0 | 0 | 0 | 0 |
| Wyoming | 0 | 0 | 0 | 0 |
| **Sum** | 76 | 41 | 19 | 13 |


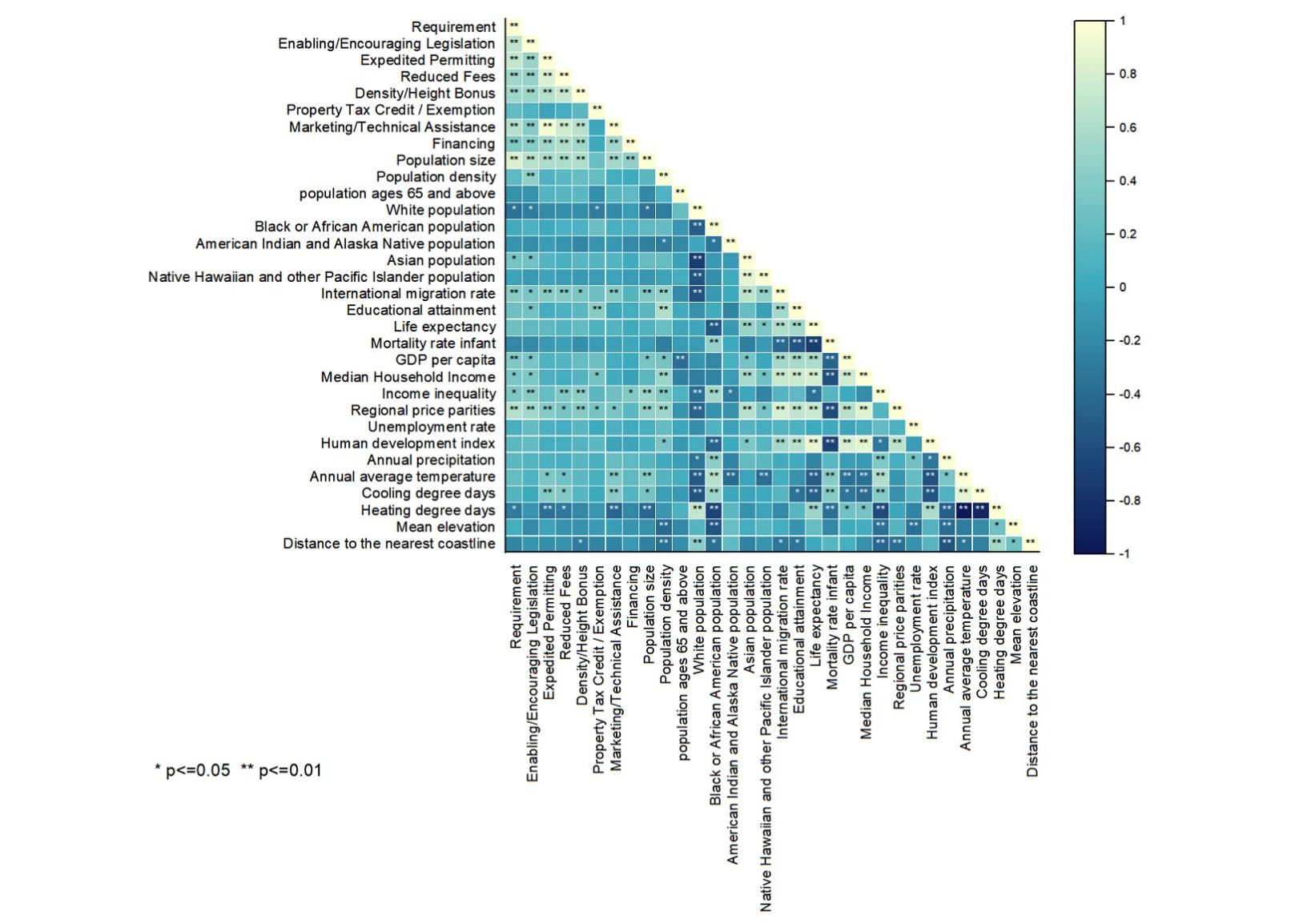


**Fig. S5.** Correlation between different demographic, socioeconomic and environmental variables and policy instruments.
